# Neoadjuvant treatment of sintilimab plus hypofractionated radiotherapy for MSI‐H/dMMR rectal cancer: A prospective, multicenter, phase Ib study

**DOI:** 10.1002/cam4.4720

**Published:** 2022-03-29

**Authors:** Xiaofen Li, Chao Fang, Xin Wang, Yongyang Yu, Ziqiang Wang, Meng Qiu

**Affiliations:** ^1^ Department of Abdominal Oncology, West China Hospital of Medicine Sichuan University Sichuan China; ^2^ Department of Gastrointestinal Surgery, West China Hospital of Medicine Sichuan University Sichuan China

**Keywords:** anti‐PD‐1 antibody, hypofractionated radiotherapy, neoadjuvant therapy, rectal cancer

## Abstract

**Background:**

Neoadjuvant radiochemotherapy followed by radical surgery is the standard treatment strategy for local advanced rectal cancer (LARC). However, the efficacy of neoadjuvant radiochemotherapy is limited, especially for patients with DNA mismatch repair‐deficient (dMMR)/microsatellite instability‐high (MSI‐H) rectal cancer. Considering the amazing therapeutic effect of immune check point inhibitors for metastatic colorectal cancer, we conduct this multicenter, phase Ib study to investigate the safety and efficacy of anti‐PD‐1 antibody, sintilimab combined with hypofractionated radiotherapy in MSI‐H/dMMR rectal cancer patients.

**Methods:**

Patients with MSI‐H/dMMR LARC will receive hypofractionated radiotherapy (5 Gy × 5) and three cycles of sintilimab 200 mg IV every 2 weeks. Radical surgery will be performed 6–8 weeks after radiotherapy. The primary endpoint is adverse reaction after neoadjuvant treatment and perioperative complications. Secondary endpoints include pathological response rate, complete resection rate, and quality of life.

**Discussion:**

This is the first study to investigate the safety and effectiveness of neoadjuvant radiotherapy combined with immunotherapy for MSI‐H/dMMR LARC. It is expected that this study will propose a brand new and effective treatment strategy for MSI‐H/dMMR LARC.

## Graphical abstract

The efficacy of neoadjuvant fluorouracil‐based radiochemotherapy is limited for rectal cancer patients with DNA mismatch repair‐deficient (dMMR)/ microsatellite instability‐high (MSI‐H) . This multicenter, phase Ib study is conducted to investigate the safety and efficacy of anti‐PD‐1 antibody, sintilimab combined with hypofractionated radiotherapy in MSI‐H/ dMMR rectal cancer. Radical surgery will be performed 6‐8 weeks after radiotherapy.

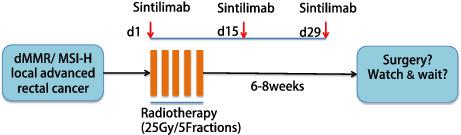

## 1 INTRODUCTION

According to the latest statistics, colorectal cancer (CRC) is the fourth most common malignancy worldwide and the second leading cause of cancer‐related deaths.^1^ Local advanced rectal cancer (LARC), defined as stage II or III rectal cancer, accounts for a considerable proportion in CRC. Due to the special anatomic location and difficulties in obtaining wide resection margins, LARC has relatively high risk of locoregional recurrence. Therefore, local radiotherapy, which is associated with a decreased local recurrence rate, is recognized as an important treatment approach for LARC. Actually, preoperative concurrent chemoradiotherapy with fluorouracil, total mesorectal excision (TME) surgery, followed by adjuvant chemotherapy has become the standard treatment for LARC.^2^

Several large phase 3 trials have demonstrated the benefit of preoperative fluoropyrimidine‐based chemotherapy with radiation, such as down staging, increasing complete resection rate, and improving pathological/clinical complete response (pCR/cCR) rate.^3^, ^4^, ^5^, ^6^, ^7^ It is generally acknowledged that pCR/cCR is associated with long‐term survival benefit and organ preservation. Hence, pCR/cCR rate is a vital factor for evaluating the effectiveness of preoperative neoadjuvant treatment in LARC. However, previous large random trials have indicated that pCR rate of neoadjuvant concurrent chemoradiotherapy with fluorouracil is very limited, ranging from 8% to 17%. In recent years, to improve the efficacy of neoadjuvant treatment, investigators have tried to intensify chemotherapy regimen, such as adding oxaliplatin, irinotecan, or total neoadjuvant therapy (TNT), in suitable patients with good physical status. Although pCR rate has improved to 19%–30%, the long‐term benefits of disease‐free survival (DFS) and overall survival (OS) have not changed.^8^, ^9^, ^10^, ^11^, ^12^, ^13^ Besides, chemotherapy intensification has increased adverse events remarkably. Thus, it is of great significance to explore a new preoperative treatment pattern to increase the possibility of anal sphincter preservation, improve long‐term survival and the quality of life.

For the past few years, immune checkpoint inhibitors, including anti‐PD‐1, anti‐PD‐L1, and anti‐CTLA4 antibodies, have shown amazing effect in multiple kinds of malignant tumors. Growing evidence supports that metastatic CRC patients with deficient mismatch repair proteins (dMMR) or high microsatellite instability (MSI‐H) are sensitive to immunotherapy.^14^, ^15^, ^16^, ^17^, ^18^ In the trial of CheckMate 142, nivolumab, an anti‐PD‐1 immune checkpoint inhibitor, shows durable response in previously treated dMMR/MSI‐H CRC patients, with the objective response rate (ORR) of 31.1%, disease control rate (DCR) of 69%, and median progression‐free survival (PFS) of 14.3 months.^15^ Given the excellent effect of immunotherapy, National Comprehensive Cancer Network (NCCN) guideline has recommended nivolumab or pembrolizumab as second‐line or more treatment for metastatic MSI‐H/dMMR CRC patients in the year of 2017. Last year, the randomized, phase 3 trial Keynote 177 have demonstrated the safety and efficacy of pembrolizumab as first‐line treatment for metastatic dMMR/MSI‐H CRC patients.^18^ Compared to chemotherapy, pembrolizumab has shown significantly longer PFS (16.5 vs. 8.2 months; hazard ratio [HR], 0.60; *p* = 0.0002).^18^ At present, immunotherapy is recommended as standard treatment for metastatic dMMR/MSI‐H CRC patients. In recent years, researchers have endeavored investigating immunotherapeutic effect in early‐stage of CRC patients with dMMR/MSI‐H. Preliminary results for NICHE trial reveals amazing neoadjuvant treatment efficacy of PD‐1 plus CTLA‐4 inhibition in early‐stage of dMMR/MSI‐H colon cancer (pCR rate: 57.1%).^19^ However, the role of immunotherapy in the neoadjuvant setting for rectal cancer is still unknown.

Additionally, increasing evidence suggests that radiotherapy, especially hypofractionated radiotherapy, has synergistic effect with immunotherapy.^20^, ^21^, ^22^, ^23^ The results of KEYNOTE‐001 trial indicate that patients treated with pembrolizumab who previously received radiotherapy have longer OS and PFS than patients who did not receive any radiotherapy (median PFS, 4.4 vs. 2.1 months; HR, 0·56, 95% CI: 0·34–0·91, *p* = 0·019; median OS, 10·7 vs. 5.3 months; HR, 0·58, 95% CI: 0·36–0·94; *p* = 0·026).^23^ The study performed by Kiess AP et al. demonstrates better survival in brain metastatic melanoma patients receiving stereotactic radiotherapy combined with ipilimumab immunotherapy, when compared to previous studies of melanoma patients treated with radiotherapy alone or ipilimumab alone.^22^

Considering good response of immunotherapy in metastatic MSI‐H/dMMR CRC patients and synergistic effect of immunotherapy combined with radiotherapy, we conduct this study to prospectively investigate the safety and efficacy of anti‐PD‐1 antibody (sintilimab) combined with hypofractionated short‐course radiotherapy as neoadjuvant treatment for dMMR/MSI‐H LARC.

## 2 STUDY DESIGN AND METHODS

### 2.1 Study design and treatment

This study is a prospective, multicenter, single‐arm, phase Ib trial to explore the safety and effectiveness of neoadjuvant treatment with sintilimab plus hypofractionated radiotherapy for dMMR/MSI‐H LARC patients. The study has obtained ethics approval and is currently ongoing in five large‐capacity hospitals in China. The main inclusion criteria are histologically confirmed local advanced rectal adenocarcinoma (no distant metastasis and clinical T stage ≥T2 on radiographic imaging), dMMR or MSI‐H status, without any anticancer treatment previously, ECOG performance status 0 or 1, age ≥18 years, and adequate organ function. Informed consent will be obtained from every subject who volunteers to participate. The main exclusion criteria include allergic or autoimmune disease history, other malignant tumor history with DFS <5 years, immunosuppressant or corticosteroid use within 2 weeks before inclusion. Complete inclusion and exclusion criteria are shown in Table 1.

**TABLE 1:** Patient inclusion and exclusion criteria

Inclusion criteria	Exclusion criteria
1. Histologically confirmed rectal adenocarcinoma 2. With DNA mismatch repair‐deficient (dMMR) or microsatellite instability‐high (MSI‐H) status, whether or not being Lynch syndrome 3. Not received any anti‐rectal cancer treatment previously; for patients with Lynch syndrome, not received any antitumor therapy about rectal cancer diagnosed this time 4. No distant metastasis except for lateral lymph nodes on thoracic and abdominal enhanced computed tomography (CT) scans; the distance between tumor's lower edge and anus within 15 cm; clinical T stage ≥T2 on high‐resolution pelvic magnetic resonance imaging (MRI) 5. Men and women ≥18 years of age 6. Eastern Cooperative Oncology Group performance status score 0 or 1 7. Adequate hematologic, hepatic, renal, thyroid, and cardiac function: hemoglobin ≥90 g/L, neutrophils ≥1500/mm^3^, platelets ≥75,000/mm^3^; aspartate aminotransferase and alanine aminotransferase ≤3.0 × upper limit of normal (ULN), bilirubin ≤1.5 × ULN; creatinine ≤1.5 × ULN, creatinine clearance ≥50 ml/min; activated partial thromboplastin time, prothrombin time and international normalized ratio ≤1.5 × ULN; serum albumin ≥28 g/L; thyroid stimulating hormone and free thyroxine within ±10% of normal levels; no obvious abnormality in electrocardiogram 8. Not received blood, blood products, and hematopoietic growth factor (e.g., granulocyte colony‐stimulating factor) within 2 weeks before inclusion 9. Informed consent form signed 10. Life expectancy of ≥3 months	1. Allergic disease history, severe hypersensitivity to drugs, antibody products or sintilimab 2. Other malignancy history with disease‐free survival <5 years, except for curative in situ cervical cancer, curative skin basal cell carcinoma and curative gastrointestinal cancer by endoscopic mucosal resection 3. Current or past history of autoimmune diseases, including but not limited to: interstitial lung disease, uveitis, enteritis, active hepatitis (HBV DNA ≥10^3^ copies/ml after regular antiviral therapy), nephritis, hyperthyroidism, and hypothyroidism 4. Immunosuppressant or corticosteroid (systemic or local) use to suppress immune function within 2 weeks before inclusion 5. Severe infection needing intravenous antibiotics, antifungal agents, or antiviral drugs, et al 6. Congenital or acquired immunodeficiency such as HIV infection; active Hepatitis B (HBV DNA ≥10^3^ copies/ml after regular antiviral therapy) 7. Having one of the following complications: massive gastrointestinal hemorrhage, gastrointestinal perforation, or obstruction; symptomatic heart diseases including unstable angina, myocardial infarction, and heart failure; uncontrollable diabetes mellitus or hypertension; uncontrollable diarrhea (interfering with daily activities although receiving adequate treatment) 8. Bleeding tendency or receiving thrombolytic or anticoagulant therapy 9. Pregnant or breastfeeding female; male and female unwilling to take any contraceptive measures 10. Psychiatric disorders that would interfere with cooperation with the requirements of the study 11. Other conditions that investigators consider not suitable for this study

**FIGURE 1:**
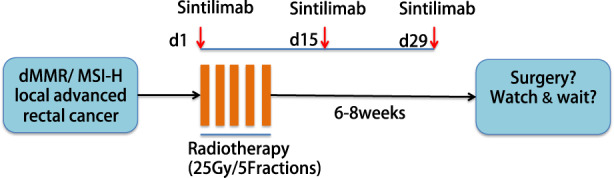
Outline of the study design

Patients eligible for participation in the trial will receive three cycles of sintilimab combined with concurrent radiotherapy. Sintilimab is administered at a dose of 200 mg intravenously every 2 weeks. Hypofractionated short‐course radiotherapy consists of 25 Gy in five fractions using intensity‐modulated radiotherapy (IMRT). Delayed radical surgery will be performed 6–8 weeks after radiotherapy. Patients who obtain cCR after careful evaluation could choose watch & wait strategy in order to preserve anal sphincter. The study design is depicted in Figure 1. Fluorouracil‐based adjuvant chemotherapy will be administered after surgery according to the NCCN guidelines.

### 2.2 Endpoints and assessments

The primary endpoint is treatment safety in terms of adverse event rate during neoadjuvant therapy and perioperative complication probability. Secondary endpoints include treatment efficacy measured with cCR/pCR rate, complete resection rate, and quality of life.

Subjects will be visited every 2 weeks to collect data about symptoms, treatment regimen, drug combination, physical and laboratory examinations, quality of life, and adverse events. Adverse events will be evaluated throughout the trial and at 28 days after treatment discontinuation or drop out, which will be graded according to the Common Terminology Criteria for Adverse Events (CTCAE) version 5.0. The quality of life will be evaluated before every cycle of sintilimab infusion, before and after 1 month of surgery, using validated questionnaires, that is European Organization for Research and Treatment of Cancer Quality of Life Core Questionnaire 30 and European Organization for Research and Treatment of Cancer Quality of Life Questionnaire‐Colorectal Cancer 29. Imaging evaluation by CT and magnetic resonance imaging (MRI) will be performed at baseline and 6–8 weeks after end of radiotherapy.

For subjects obtaining cCR after neoadjuvant treatment, organ preservation strategy should be adopted prudently after careful assessment with digital examination, endoscopy, chest CT, abdominal CT, and pelvic MRI. A multidisciplinary team evaluation is recommended. The cCR is defined as the absence of any palpable tumor at digital rectal examination; no visible lesion at endoscopy except a flat scar, telangiectasia or mucosa whitening and negative biopsies from the scar; and residual tumor absence on imaging with MRI or endoscopic rectal ultrasound (ERUS).^24^

The expression of four mismatch repair proteins (MLH1, MSH2, MSH6, and PMS2) is determined locally by immunohistochemical analysis. And if one or more of the four proteins is absent, the tumor status is defined as dMMR. MSI status is determined locally by polymerase chain reaction (PCR) analysis of five tumor microsatellite loci (NR‐21, NR‐24, BAT‐25, BAT‐26, and MONO‐27). And if two or more of the five loci are present, the tumor status is classified as MSI‐H.

### 2.3 Sample size and statistical analyses

As the primary end point of this phase Ib study is treatment safety, no formal sample size calculation is performed. Based on the low incidence of dMMR/MSI‐H LARC and expected number of patients in our research institutes, we plan to enroll 20 subjects in total. SPSS Statistics version 22.0 (IBM) will be used to perform all the statistical analyses.

## 3 DISCUSSION

Preoperative fluoropyrimidine‐based chemotherapy with concurrent long‐course radiation is the standard neoadjuvant treatment for LARC patients. But as we have stated in the introduction part, neoadjuvant chemoradiotherapy has limited effectiveness and unsatisfactory adverse events. In recent years, several large‐scale trials have demonstrated that hypofractionated short‐course radiotherapy with/without chemotherapy followed by delayed surgery can obtain higher pCR rate and lower radiation‐related adverse reactions.^25^, ^26^, ^27^ Nonetheless, the efficacy is still unsatisfactory, especially for dMMR/MSI‐H patients. As has been reported, colorectal cancer patients with dMMR/MSI‐H status are relatively resistant to fluoropyrimidine‐based chemotherapy but sensitive to immunotherapy.^18^, ^28^ Inspired by synergistic effect of immunotherapy with hypofractionated short‐course radiotherapy, we have designed this trial to explore the efficacy and safety of neoadjuvant immunotherapy combined with hypofractionated radiotherapy followed by delayed surgery in dMMR/MSI‐H LARC patients.

At present, there are several trials investigating the value of neoadjuvant immunotherapy in rectal cancer. For LARC patients with microsatellite stable (MSS), the study performed by Shamseddine A. et al. reveals promising efficacy of short‐course radiotherapy followed by mFOLFOX6 chemotherapy plus avelumab (anti‐PD‐L1 antibody), with a pCR rate of 25% (3/12).^29^ In the single‐arm, phase II study conducted by Lin Z. et al., LARC patients receiving short‐course radiotherapy with capecitabine and oxaliplatin chemotherapy plus camrelizumab (anti‐PD‐1 antibody) have a relatively high pCR rate of 48.1% (13/27), including 46.2% (12/26) for patients with proficient mismatch repair (pMMR) status and 100% (1/1) for dMMR patient.^30^ Preliminary results of VOLTAGE trial where 41 LARC patients receive long‐course radiotherapy with concurrent capecitabine chemotherapy followed by five cycles of nivolumab (anti‐PD‐1 antibody), shows a pCR rate of 30% in pMMR patients (11/37) and 60% in dMMR patients (3/5) with mild toxicities.^31^ Although these results show favorable safety and efficacy of preoperative immunotherapy, most patients in the trials are MSS/pMMR, with only a few MSI‐H/dMMR patients. Besides, their treatment approaches contain chemotherapy as well. The phase 2 trial conducted by Hu H. et al. reveals favorable pCR rate of neoadjuvant toripalimab with or without celecoxib in MSI‐H locally advanced colorectal cancer patients. But this trial mainly enrolls colon cancer patients (30/36), with only six rectal tumors.^32^ In contrast, our study will only enroll MSI‐H/dMMR LARC patients and get rid of chemotherapeutic agents. We believe this study will illustrate the safety and effectiveness of radiotherapy combined with immunotherapy in MSI‐H/dMMR LARC patients, and propose a brand new treatment approach for this specific group of patients.

There are several limitations in our study, including a small sample size, lack of a control group, and uncertain pathological examination after neoadjuvant treatment in patients who choose watch & wait strategy.

## CONFLICT OF INTEREST

All the authors declare no potential competing interest.

## AUTHOR CONTRIBUTION

MQ is the principal investigator. MQ and ZW are responsible for the trial design.

XL is responsible for recruitment and patient information. CF and YY are responsible for patient follow‐up. XW is responsible for statistical analysis. XL, CF, ZW, and MQ drafted and revised the manuscript. All authors reviewed the manuscript and approved to the submission.

## ETHICS STATEMENT

This study has been approved by West China Hospital of Sichuan University Clinical Trial Ethics Committee. All candidate subjects must sign written informed consent before enrollment.

## PERMISSION TO REPRODUCE MATERIAL FROM OTHER SOURCES

Not applicable.

## TRIAL REGISTRATION

This trial is registered at ClinicalTrials.gov (NCT04636008).

## Data Availability

Data sharing is not applicable to this article as no new data were created or analyzed in this study.
